# Extracting microtentacle dynamics of tumor cells in a non-adherent environment

**DOI:** 10.18632/oncotarget.22874

**Published:** 2017-12-04

**Authors:** Eleanor C. Ory, Desu Chen, Kristi R. Chakrabarti, Peipei Zhang, James I. Andorko, Christopher M. Jewell, Wolfgang Losert, Stuart S. Martin

**Affiliations:** ^1^ Department of Physics, IPST, and IREAP, University of Maryland, College Park, MD 20742, USA; ^2^ Marlene and Stewart Greenebaum NCI Cancer Center, University of Maryland School of Medicine, Baltimore, MD 21201, USA; ^3^ Program in Molecular Medicine, University of Maryland School of Medicine, Baltimore, MD 21201, USA; ^4^ Fischell Department of Bioengineering, University of Maryland, College Park, MD 20742, USA; ^5^ Department of Microbiology and Immunology, University of Maryland School of Medicine, Baltimore, MD 21201, USA; ^6^ Department of Physiology, University of Maryland School of Medicine, Baltimore, MD 21201, USA; ^7^ United States Department of Veterans Affairs, Baltimore, MD 21201, USA

**Keywords:** microtentacles, cytoskeleton, image analysis, circulating tumor cells, mechanobiology

## Abstract

During metastasis, tumor cells dynamically change their cytoskeleton to traverse through a variety of non-adherent microenvironments, including the vasculature or lymphatics. Due to the challenges of imaging drift in non-adhered tumor cells, the dynamic cytoskeletal phenotypes are poorly understood. We present a new approach to analyze the dynamic cytoskeletal phenotypes of non-adhered cells that support microtentacles (McTNs), which are cell surface projections implicated in metastatic reattachment. Combining a recently-developed cell tethering method with a novel image analysis framework allowed McTN attribute extraction. Full cell outlines, number of McTNs, and distance of McTN tips from the cell body boundary were calculated by integrating a rotating anisotropic filtering method for identifying thin features with retinal segmentation and active contour algorithms. Tethered cells behave like free-floating cells; however tethering reduces cell drift and improves the accuracy of McTN measurements. Tethering cells does not significantly alter McTN number, but rather allows better visualization of existing McTNs. In drug treatment experiments, stabilizing tubulin with paclitaxel significantly increases McTN length, while destabilizing tubulin with colchicine significantly decreases McTN length. Finally, we quantify McTN dynamics by computing the time delay autocorrelations of 2 composite phenotype metrics (cumulative McTN tip distance, cell perimeter:cell body ratio). Our automated analysis demonstrates that treatment with paclitaxel increases total McTN amount and colchicine reduces total McTN amount, while paclitaxel also reduces McTN dynamics. This analysis method enables rapid quantitative measurement of tumor cell drug responses within non-adherent microenvironments, using the small numbers of tumor cells that would be available from patient samples.

## INTRODUCTION

The study of circulating tumor cells is a rapidly growing field of research and diagnostics [1, 2]. Considering that 90% of cancer fatalities are the result of metastasis [3], tumor cell survival in circulation is a rate-limiting step in the metastatic cascade. Thus circulating tumor cells (CTC) present a valuable opportunity for understanding patient prognosis and possible strategies to reduce dissemination. Already, research has demonstrated that CTCs can be detected early during cancer disease progression and demonstrated valuable prognostic value for distant disease free survival and potential superiority over current imaging methods [1, 4–8]. Furthermore, a higher CTC count is correlated with a poorer patient prognosis [1, 2, 5]. Most recently, results from a prospective clinical trial show that CTC’s appear in the bloodstream an average of 6 months prior to detection on a PET/CTC scan [9]. The vast majority of primary breast cancers are carcinomas, where sarcomas account for less than 1% [10] and lymphomas less than .5% [11]. According to the American Cancer Society, while the survival rates of breast cancer stages 0-1 are approximately 100% and 93% respectively, metastasized breast cancer has only a 22% survival rate (ACS). Most breast cancer metastases are thought to spread by circulating through the bloodstream before colonizing distant tissue. Given that the vast majority of breast cancer cells are epithelial, understanding how these adherent cells behave in a non-adherent environment is a critical and understudied question. Refining our understanding of CTC characteristics and reattachment mechanisms represents an underutilized approach for improving patient diagnostics and drug therapies.

One challenge in improving the treatments of metastatic breast cancer is the highly variable latency time where cancer cells may stay dormant for years or as long as decades prior to detection [12–15]. Historically, the incredibly low concentration of CTCs, which are as rare as 1 CTC in 100 million to 1 billion blood cells, has posed a technological hurdle to further research and improve our understanding of the role CTCs play in metastasis [2, 16]. Recently, an abundance of emerging technologies has improved the efficacy and efficiency of capturing and segregating CTCs [1, 2, 16–18]. It is now feasible to capture 10 CTCs or more from a typical patient blood sample size [19]. Although CTCs can now be extracted from the bloodstream, further characterization of the cells is very limited, particularly characterizing cells in their native environment of suspension. Currently, the only FDA-approved downstream analysis (CellSearch) simply enumerates total number of chemically-fixed CTCs or the presence of particular biomarkers using immunostaining [1]. Most image analysis techniques for suspended cells have focused on detecting and measuring immunofluorescence levels for a particular biomarker. Since tumor cells in a non-adherent environment float freely, they move notably due to thermal fluctuations or residual fluid flows. Without confining boundaries, dynamics will be three-dimensional. These fluctuations have mostly prevented three-dimensional imaging and time-lapse single cell imaging of CTC shape and dynamics.

Currently, little is known about which circulating tumor cells succeed in surviving the blood-stream and ultimately forming metastases [20]. However, one likely morphological phenotype of cell reattachment that was found in numerous metastatic breast tumor cell lines is the presence of microtentacles, McTNs [21, 22]. McTNs are tubulin-based protrusions found in tumor cells in a non-adherent environment; McTN positive cells reattach to endothelial cells, and are more efficiently retained in lung capillaries, so McTNs are a promising indicator for evaluating reattachment potential [21, 23]. A higher McTN number is found in more invasive breast cancer cell lines [21]. Mcf10 PTEN-/- cells are McTN-positive and have dormancy characteristics such as anoikis resistance and arrested cell cycle in suspension [24, 25]; when injected into mice, mcf10 PTEN-/- cells persist, but don’t grow into large tumors [26]. Current evidence has not demonstrated a role for McTNs in tumor growth, but instead support a model where McTNs promote tumor cell reattachment during metastasis [23]. Also, molecular mechanisms that support McTNs are associated with increased metastasis and poor patient prognosis [27].

As a result of both standard imaging and analysis techniques, there were previously no reliable methods to capture the thin McTN structures. Most microfluidic systems for CTCs allow cells to float out of the field of view quickly, thus limiting data to snapshots of CTCs as they passed by the imaging area. Most prior work on McTNs relied on manual scoring of the presence or absence of McTNs. Here we use a novel cell tethering technique, recently developed and validated by the Martin group that allows us to hold a cell in place within the field of view of the microscope over long time periods, and thus enable extended time-lapse imaging of McTN behavior [28]. To analyze these much larger datasets we developed image analysis approaches to quantify McTN number, McTN tip distance, and McTN dynamics. Overall, in order to improve techniques for studying tumor cells in a non-adherent environment we did the following: (1) Measured drift and present quantitative evidence that the tethering method improves the ability to visualize McTNs. (2) Developed tools enabling quantification of morphology in tumor cells in a non-adherent environment including: area of the cell body, variance in the cell body area, ratio of the full cell perimeter to the cell body perimeter, distance of McTN tips from cell body perimeter, number of McTN tips, and cumulative tip distance. (3) Demonstrate feasibility of distinguishing phenotype characterization by showing that the length of McTNs in paclitaxel-treated cells is a greater contributor to overall McTN phenotype compared to number of McTN tips. (4) Present evidence that drug treatments change dynamics of morphology.

## RESULTS

**Figure 1:**
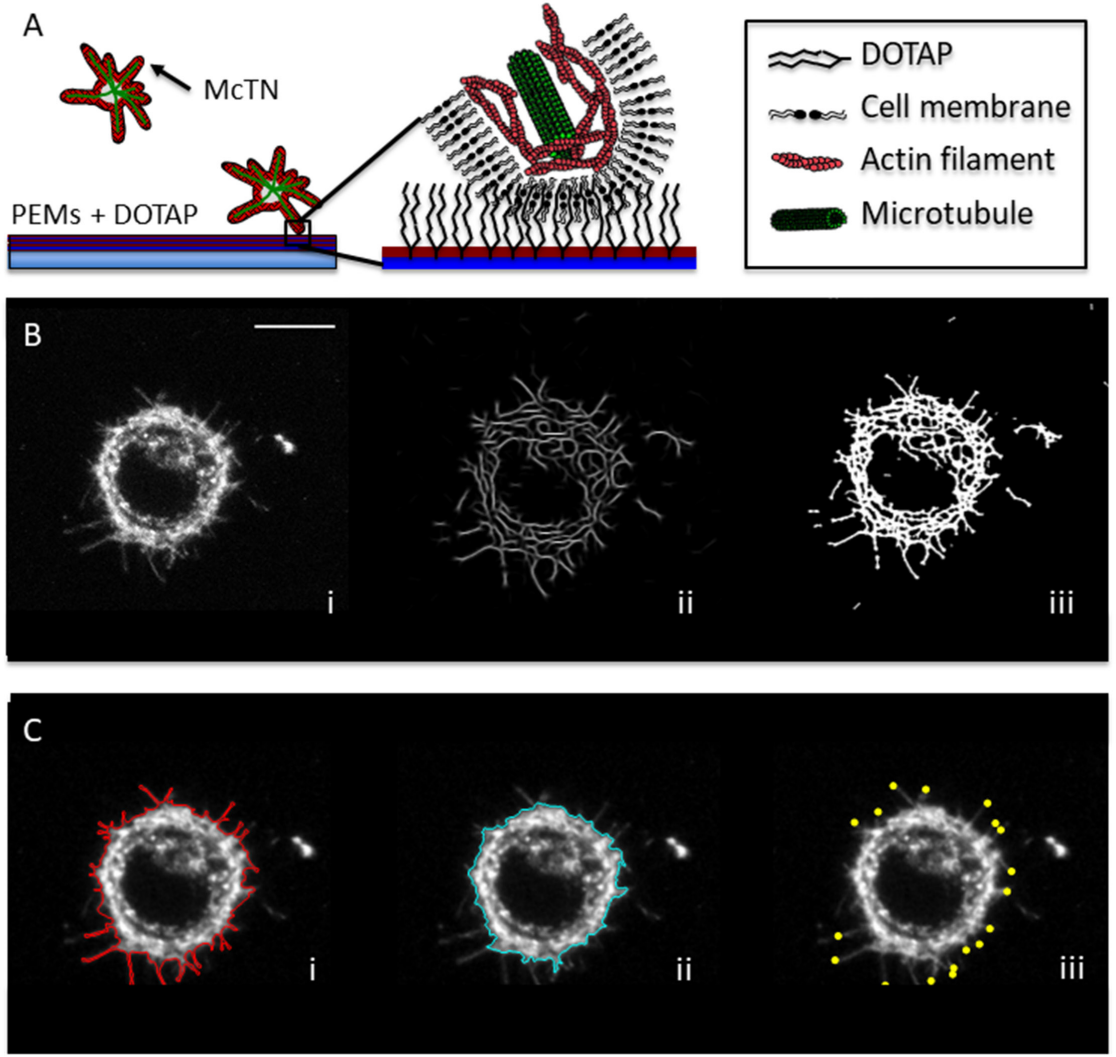
Lipid tethering and image analysis techniques define morphological attributes quantitatively **(A)** Tumor cells cannot form adhesions on surfaces coated with polyelectrolyte multilayers (PEMs), but integration of a lipid (DOTAP) into the upper PEM surface promotes hydrophobic tethering with the cell membrane. Tethered cells maintain non-adherent behaviors, like the formation of McTNs that are supported by microtubule extension and suppressed by actin contraction. **(B)** Image analysis methods for full cell outline enhance McTN visualization by taking the maximum intensity profile of a 5 stack z-projection for a particular time point (i) and undergoing several iterations of anisotropic filtering (ii) before thresholding the results into a binary image (iii). **(C)** Attributes derived from image analysis consists of outline of the full cell including McTNs (i), outline of the cell body’s boundaries excluding McTNs (ii) and tips of McTNs (iii) derived from maximum local curvature of skeletonization of full cell outline (scalebar = 10μm).

As the study of CTCs progresses and technologies to capture viable CTCs emerge, there is a growing need for approaches to analyze cell shapes and dynamics for tumor cells in a non-adherent environment. We have previously shown that tethering suspended cells is an effective technique for studying tumor cells in a non-adherent environment [28]. The tethering technique attaches a small part of the cell’s membrane to a surface while allowing the cell to retain its non-adherent characteristics (Figure 1A). Here, we develop image analysis techniques to quantitatively measure the advantages of cell tethering, present new McTN metrics, make new distinctions on McTN structure based on these metrics, as well as demonstrating the dynamics in response to tubulin-targeting drugs for tethered cells.

### Anisotropic filter allows us to capture outline of microtentacles

To test the ability of our image analysis to detect McTNs, microfluidics chambers were prepared with 2 different surface treatments. In order to compare free-floating cells with tethered cells, we used MDA-436 and MDA-231 cells, mesenchymal triple-negative cell lines with a high metastatic potential known to form McTNs [29]. For free-floating cells, microfluidic chambers were coated with pluronic F-127, a generally cytophobic coating to prevent cell attachment. For tethered cells, microfluidic chambers were coated with cytophobic polyelectrolyte multilayers (PEMs) followed by a lipophilic coating of DOTAP to hydrophobically bind the lipid membrane while maintaining free-floating cell behaviors (Figure 1A) [28].

Combining several existing image analysis techniques, we devised an image analysis framework for finding both the McTNs and the cell body. Currently, there exist image analysis techniques that are optimized for attached globular shapes as well as techniques for stress fibers [30, 31]. To identify the faint, fibrous structures of McTNs, we convolved the images with a rotating anisotropic filter, taking the output of the rotating anisotropic filter, and repeating the rotating anisotropic filtering for a variable number of several iterations prior to thresholding (Figure 1B). Combined with a separate analysis approach to extract the cell body boundary as detailed in the Methods section, we were able to extract pertinent attributes of suspended cells including, cell body outline (cell outline without McTNs), outline of the cell shape that includes McTNs, and a measurement of McTN tips (Figure 1C).

### Tethering prevents cells from drifting and improves visualization of microtentacles

**Figure 2:**
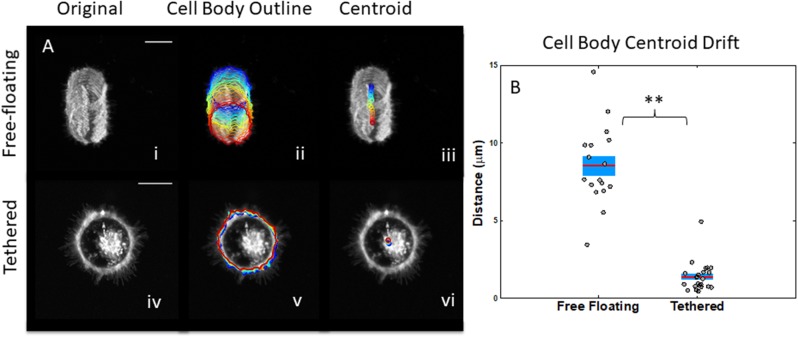
Measurements of lateral cell drifting to compare free-floating cells to tethered cells **(A)** Time projection profile of maximum intensity z-stacks for free-floating cells (top) and tethered cells (bottom). Overlays of cell body outline (ii) from initial (blue) to final (red) time points for free-floating cells (top) and tethered cells (bottom). Centroid of cell body (iii) from initial (blue) to final (red) time points for free-floating cells (top) and tethered cells (bottom). **(B)** Average total distance traveled by centroid of cell body for free-floating cells is greater than tethered cells. Horizontal bar represents average across cells; shaded area, SEM; and individual dots, mean per cell (scalebar = 10μm). ^*^P<0.05; ^**^P<0.001 t-test.

Previous research demonstrated that tethered cells stay attached to the surface after several washes better than free-floating cells [28]. However, these studies were unable to quantify the ability of lipid tethers to decrease cellular drift across the microfluidic surface. In this study, our image analysis techniques allowed us to determine the drift of individual free-floating and tethered cells qualitatively by looking at the maximum intensity projections over time, overlays of cell body boundary as a function of time, and overlays of the centroid of the cell body boundary as a function of time (Figure 2A). Computing total distance traveled by the cell body’s centroid, we demonstrated quantitatively that tumor cells in a non-adherent environment have significantly more lateral drifting than tethered cells (Figure 2B) where the t-test p-value was 9x10^-15^ and ks-test p-value was 5x10^-9^.

**Figure 3:**
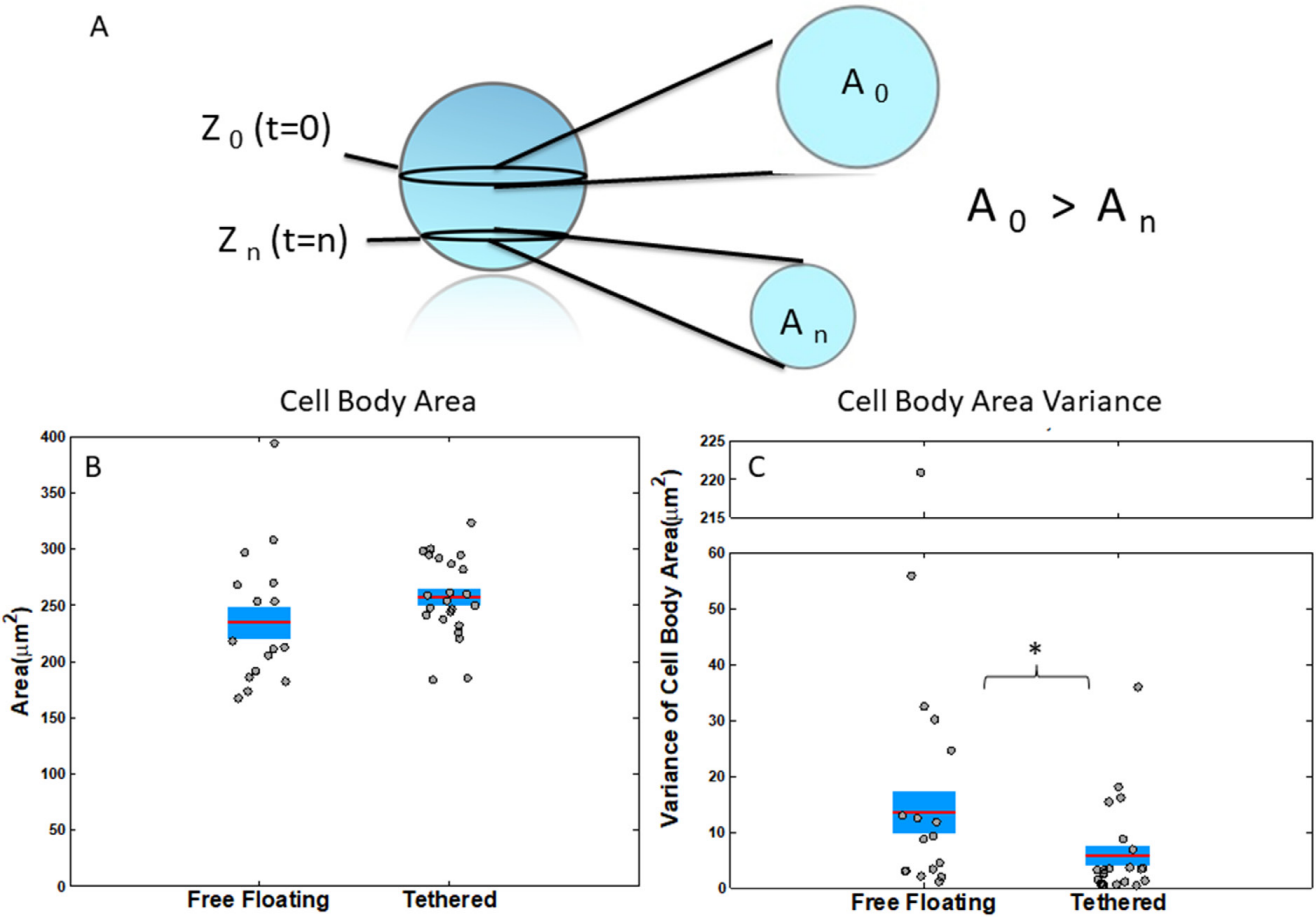
Measurements of cell body attributes for free-floating and tethered cells **(A)** Schematic of cell’s cross-sectional area at different z-planes. Cross-sectional area is largest, when the slice crosses the center. **(B)** Average cell body cross-sectional area of free-floating and tethered cells has no significant difference p=.14. **(C)** Variance of cell body area over time for free-floating and tethered cells ks-test p=.0496. Horizontal bar represents average across cells; shaded area, SEM; and individual dots, mean per individual cell across time series. ^*^P<0.05; ^**^P<0.001 Kolmogorov-Smirnov test.

Using binary cell body image results from image analysis, the average cell body area over time across all cells and cell body area variance per cell across time was computed (Figure 3A). Results showed that cell body area of free-floating cells was potentially slightly smaller than tethered cells but not significantly with a t-test p-value of .14 (Figure 3B). Because all z-stacks were centered at the largest part of the cell and because the z-stack thickness is thinner than the cell diameter, the slightly larger cell body area for tethered cells may indicate that free-floating cells were also drifting vertically to smaller cross-sectional cell areas in the z-plane (Figure 3A). Furthermore, free-floating cells had a significantly higher variance (ks-test p=.0496) in the cell body area which further substantiated that the cells were moving slightly out of plane along the z-axis (Figure 3C).

**Figure 4:**
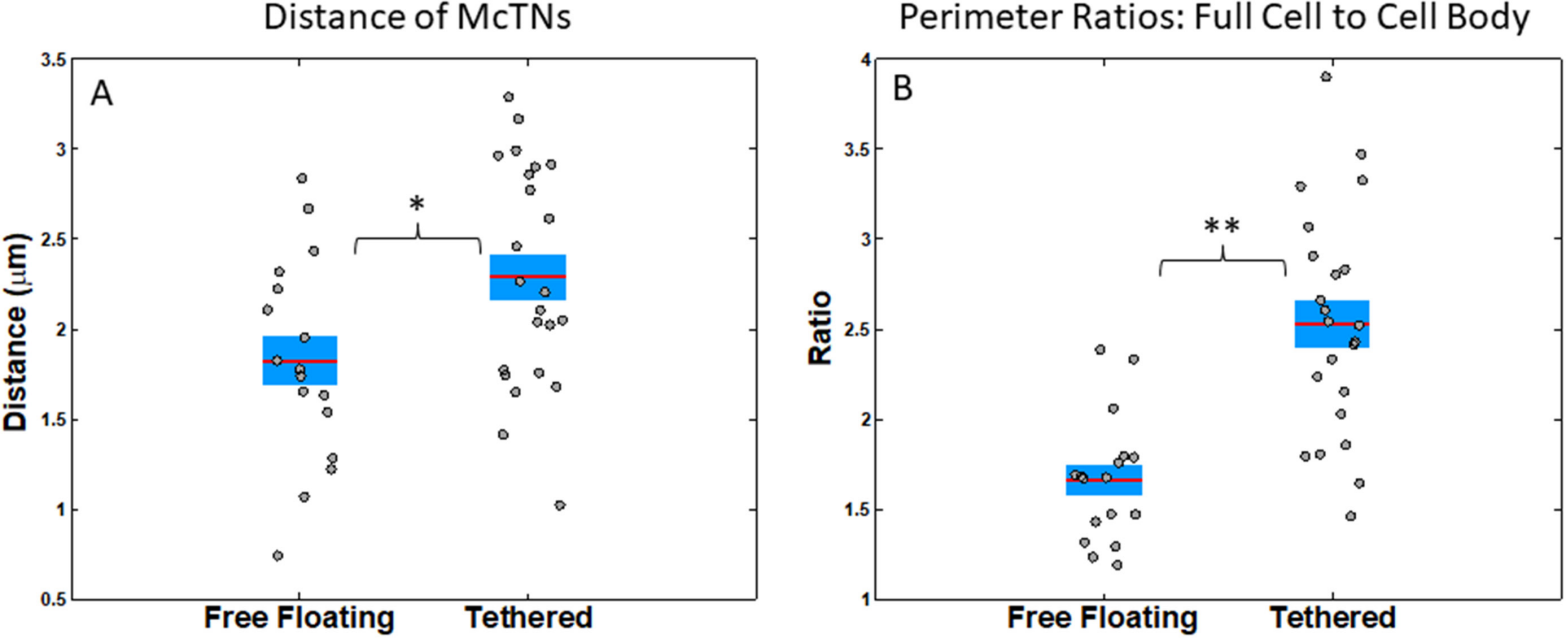
Statistics of free-floating versus tethered cells’ metrics suggest that tethered cells allow better visualization of microtentacles **(A)** Average distance of McTN tips from cell body boundary for free-floating and tethered cells (p=0.02). **(B)** Average ratio of perimeter of the full cell outline to cell body outline for free-floating and tethered cells (t-test p=8.9944e-06). Horizontal bar represents average across cells; shaded area, SEM; and individual dots, mean per cell across time series. ^*^P<0.05; ^**^P<0.001 t-test.

We extended our image analysis technique to further characterize McTNs quantitatively and apply McTN metrics for both tethered and free-floating cells. From our image analysis code, we estimated McTN length by measuring the distance of the McTN tip from the nearest cell body boundary point. We found that tethered cells have a larger average distance from McTN tip to cell body boundary than free-floating cells with a t-test p-value.02 (Figure 4A). Another way we measured total McTN phenotype was by taking the ratio between the full cell perimeter and the cell body perimeter; this allowed us to compare McTN perimeter, while normalizing by cell size. Tethered cells exhibited a higher average ratio of full cell perimeter to cell body perimeter than free-floating cells (t-test p=9x10^-6^) suggesting that tethering allows one to better capture McTNs than the free-floating technique (Figure 4B). For the interpretation of this analysis, we assume that the average McTN length and number should be the same for tethered as for free-floating cells. While this is consistent with prior published work [28], prior studies did not have image analysis capabilities and consequently not the accuracy of our quantitative analysis.

### Image analysis captures microtentacles qualitatively and quantitatively with drug treatments

**Figure 5:**
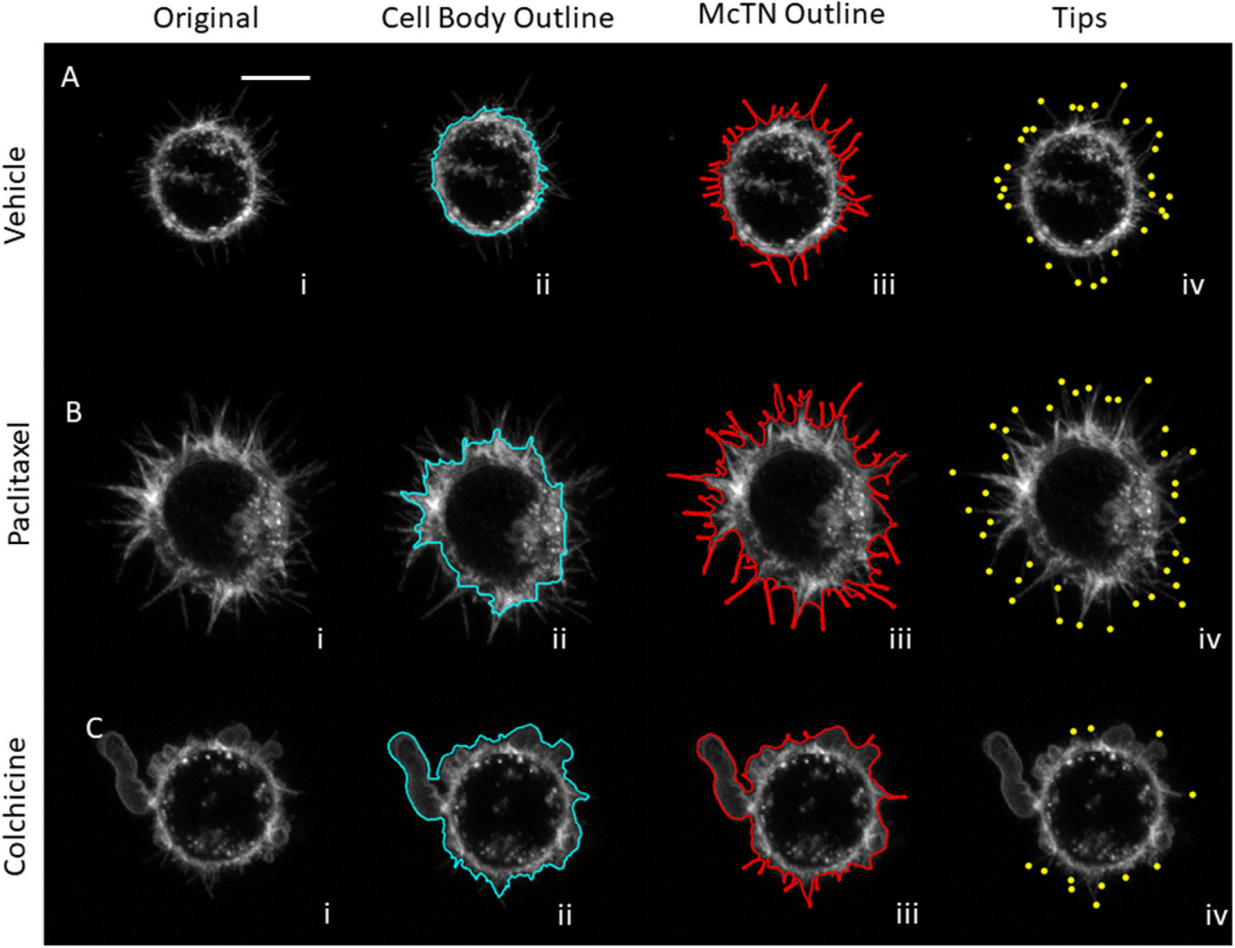
Image analysis attributes for microtubule-targeting drug treatments for MDA-MB-436 cells **(A)** Max projection of z-stack for MDA-436 cells treated with vehicle (i) is analyzed to find cell body boundary (ii), outline of full cell, (iii) and tips of McTNs (iv). **(B)** Max projection of z-stack for MDA-436 cells treated with 10 μg/mL paclitaxel (i) analyzed for cell body boundary (ii), outline of full cell, (iii) and tips of McTNs (iv) shows increase in McTNs. **(C)** Max projection of z-stack for MDA-436 cells treated with 125 μM colchicine (i) analyzed for cell body boundary (ii), outline of full cell, (iii) and tips of McTNs (iv) shows a decrease in McTNs (scalebar = 10μm).

Once we determined that we were able to visualize and effectively quantify more McTNs with the combination of cell tethering and image analysis, we used tethered cells to quantify, for the first time, the effects of tubulin-targeting drugs on McTNs. For our first analysis, we selected drugs paclitaxel and colchicine, which have previously demonstrated the ability to enhance or diminish McTNs by respectively stabilizing or destabilizing the microtubules that form McTNs. On tethered surfaces, we calculated the attributes of cell body boundary, full cell outline, and McTN tips for cells treated with vehicle or 0.1% DMSO, 10 μg/mL paclitaxel, and 125 μM colchicine for both MDA-436 and MDA-231 cells (Figure 5 and Supplementary Figure 3).

**Figure 6:**
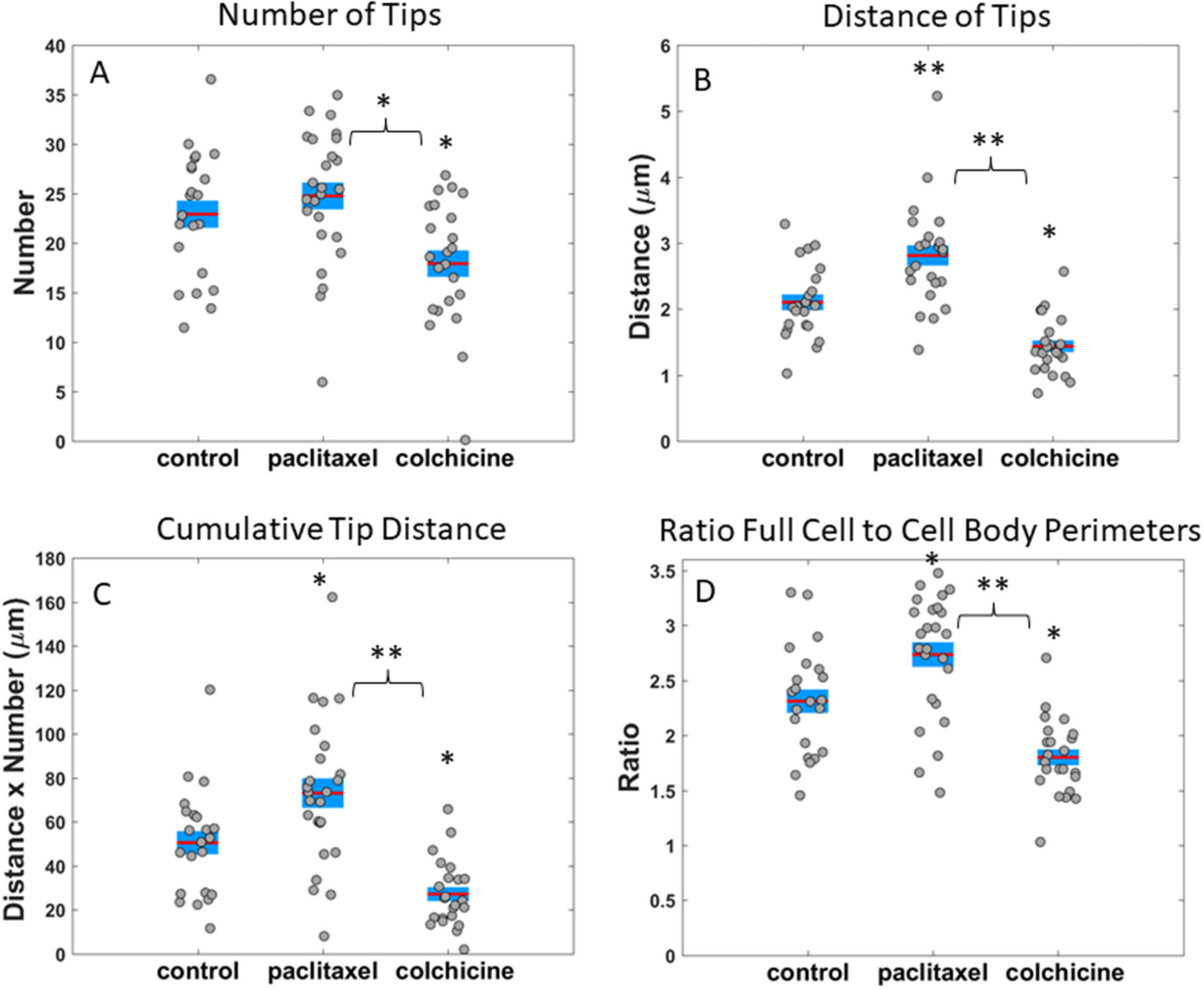
Measurements of microtentacle attributes in MDA-MB-436 cells for different drug treatments **(A)** Average number of McTN tips for cells treated with vehicle, 10 μg/mL paclitaxel, and 125 μM colchicine. **(B)** Average distance of McTN tips from cell body boundary for cells treated with vehicle, 10 μg/mL paclitaxel, and 125 μM colchicine. **(C)** Average cumulative tip distance, calculated by multiplying total number of McTN tips by the average distance of McTN tip from cell body per frame, is shown for cells treated with vehicle, 10 μg/mL paclitaxel, and 125 μM colchicine. **(D)** Ratio of perimeters for full cell outline to cell body boundary is shown for cells treated with vehicle, 10 μg/mL paclitaxel, and 125 μM colchicine. Horizontal bar represents average across cells; shaded area, SEM; and individual dots, mean value per cell across time series. ^*^P<0.05; ^**^P<0.001 ANOVA test.

Deriving metrics from our image analysis attributes, we quantified the differences between cells treated with vehicle, 10 μg/mL paclitaxel, and 125 μM colchicine. For both MDA-436 and MDA-231 cells, colchicine-treated cells had significantly fewer McTNs than the vehicle with an anova p-value of 0.03 and.007 respectively (Figure 6A and Supplementary Figure 4A). However, there was no significant difference in the number of McTNs between vehicle and paclitaxel treated cells (Figure 6A and Supplementary Figure 4A). Looking beyond McTN number per cell, the distance of the McTN tips from the cell body boundary was significantly higher in paclitaxel-treated cells compared to vehicle-treated cells for both cell lines with an anova p-values of 4.6e-04 and 1.2e-04 (Figure 6B and Supplementary Figure 4B). In addition to the significant decrease in McTN number with colchicine, McTN tip distance was also significantly lower in colchicine treated cells (anova p=1.1e-09 and 3.9e-08) than in vehicle treated cells (Figure 6B and Supplementary Figure 4B). A cell may be perceived as having a stronger McTN phenotype either by increasing the number of McTNs or by increasing the length of McTNs. Currently, it is unknown whether number of McTNs, length McTNs or both are the most critical phenotypes for reattachment. In order to look at McTN phenotype as a whole, we introduced two aggregate McTN phenotype metrics. One way that we measured the aggregate McTN phenotype, was to multiply the number of McTNs by the average distance of McTN tips from the cell body boundary per frame per cell; in essence, the cumulative McTN tip distance for an entire cell within a given imaging frame. The cumulative tip distance allows short McTNs to be included in the measurement while essentially weighing their contribution as less than longer McTNs. Paclitaxel-treated cells for both MDA-436 and MDA-231 had a significantly higher cumulative tip distance (ANOVA p=0.011 and.003) compared to vehicle (Figure 6C and Supplementary Figure 4C). Colchicine treated cells, on the other hand, had a significantly lower cumulative tip distance (anova p=9.8e-03 and.039) compared to control (Figure 6C and Supplementary Figure 4C). An additional metric we utilized to measure overall McTN phenotype was to take the ratio between the full cell outline and cell body outline; this method has the advantage of including the entire length and curve of McTNs unlike the cumulative tip distance linear metric, but was still normalized to the size of the cell body. For the ratio of outlines metric (Figure 6D and Supplementary Figure 4D), we found that paclitaxel treated cells had a higher ratio than vehicle for MDA-436 cells only (anova p=9.8e-03 vs.14) while colchicine had a lower ratio than vehicle for both MDA-436 and MDA-231 cells (p=1.9e-03 and.015). Cumulative tip distance (Figure 6C and Supplementary Figure 4C) and ratio (Figure 6D and Supplementary Figure 4D), were both more robust than average tip number (Figure 6A and Supplementary Figure 4A) or average tip distance (Figure 6B and Supplementary Figure 4B) metrics.

### Dynamic analysis of shapes measures stability of drug treatments

**Figure 7:**
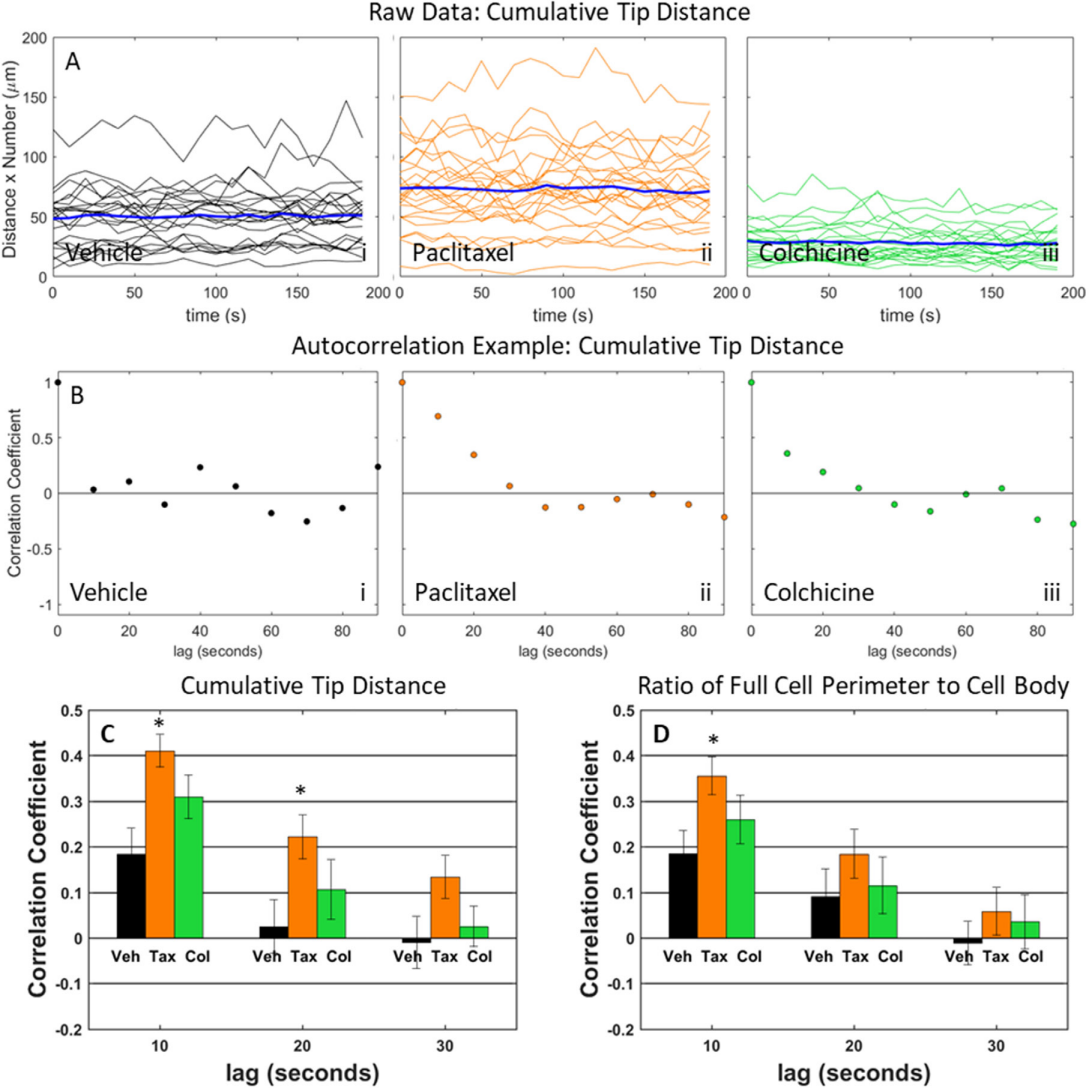
Dynamic behavior in MDA-MB-436 cells is assessed by analyzing cumulative tip distance and the ratio of full cell perimeter to cell body perimeter **(A)** Time traces or cumulative tip distance for individual cells treated with vehicle (i), 10 μg/mL paclitaxel (ii), and 125 μM colchicine (iii). Bold blue time trace is average cumulative tip distance over all individual cells. **(B)** Example autocorrelation traces of cumulative tip distance for individual cells treated with vehicle (i), 10 μg/mL paclitaxel (ii), and 125 μM colchicine (iii). **(C)** Fluctuations of cumulative distance is shown by computing the autocorrelation coefficient at time lags 0 to 30 seconds for cells treated with vehicle, 10 μg/mL paclitaxel, and 125 μM colchicine. **(D)** Fluctuations of ratio between full cell outline and cell body boundary is shown by computing the autocorrelation coefficient at time lags 0 to 30 seconds for cells treated with vehicle, 10 μg/mL paclitaxel, and 125 μM colchicine.

Our shape measures that capture McTN composite phenotype, allow us to measure dynamic fluctuations in response to drug treatment. A graph of the cumulative tip distance as a function of time, with separate traces for each individual cell, shows a large cell to cell variation in addition to the variations with drug treatment (Figure 7A and Supplementary Figure 5A). The mean cumulative distance for each drug treatment as a function of time, however, was relatively stable suggesting a normal distribution (Figure 7A and Supplementary Figure 5A). To measure characteristic timescales of fluctuations, the autocorrelation coefficient of the cumulative tip distance was computed for time intervals ranging from 10 s to 90 s apart for each individual cell (Figure 7B and Supplementary Figure 5B). For each cell type and drug condition, the mean temporal autocorrelation coefficient was computed for three time intervals: 10 s, 20 s, and 30 s across all cells for both the cumulative tip distance and ratio of full cell outline to cell body outline (Figure 7C-7D and Supplementary Figure 5C-5D). For MDA-436 cells, the data showed that up to 20 seconds apart, cells treated with paclitaxel had higher autocorrelation (and therefore less fluctuations) than the vehicle for cumulative tip distance (Figure 7C). Consistent with the autocorrelation coefficient of cumulative tip distance, the autocorrelation coefficient of the ratio of full cell outline to cell body outline also showed higher autocorrelation in cells treated with paclitaxel compared to control as far as 10 seconds apart (Figure 7D). Likewise, MDA-231 cells showed significantly less morphological fluctuations for paclitaxel-treated cells compared to colchicine-treated cells for cumulative tip distance at 10s apart (Supplementary Figure 5C) and ratio of full cell outline to cell body outline at 30s apart (Supplementary Figure 5D).

## DISCUSSION

Current techniques for McTN analysis require double-blinded studies where McTNs are manually enumerated. Such techniques are time consuming and potentially lack uniformity. Additionally, previous manual studies formally defined McTNs as narrow protrusions at least the size of the cell body radius evaluated by the qualitative perception of the user [29]. Rather than assign an arbitrary McTN distance cutoff, the new technique uniformly applies a max curvature measurement. For the blebbing morphology of colchicine, most of the large protrusions’ lower curvature appropriately does not register as McTNs. Although, the automated method may occasionally be more likely to have false positives for McTN counts (ie colchicine treatment), this image analysis is still able to show clearly distinct populations in the number of McTNs in a statistically significant manner. For the first time, we are able to evaluate McTN number automatically and systematically with multiple quantitative measurements of cell phenotype including distance of McTN tips from the cell body and number of McTNs. Previous research based on qualitative assessment of positive or negative McTN phenotype concluded that paclitaxel appeared to increase mostly the length of McTNs and a combination of latrunculin and paclitaxel appeared to increase the number of McTNs [22, 29]. While one previous study manually counting the number of McTN tips found an increase in the number of tips in cells treated with paclitaxel compared to control, the study did not include shorter protrusions nor exclude cells with significant drift [28]. In this study, we were able to verify quantitatively that paclitaxel increases the length of McTNs rather than the number of McTNs. Such distinctions may pave the way towards determining whether the number of McTNs or the length in McTNs more strongly affect CTC reattachment.

Our results demonstrate statistically-significant, morphological differences using as few as 19 non-adhered tumor cells, which could increase feasibility with the limited number of CTCs currently recoverable with existing isolation technologies [17]. Alternative analysis methods, like flow cytometry, require tens of thousands of cells. Attempts to expand patient tumor cells through mouse xenografts of either CTCs or primary tumor cells is often unsuccessful, leading to significant loss of patient population representation [32]. Moreover, patient-derived xenografts can require greater than 6 months to establish and more than 2 years for a complete drug study [33, 34]. The tethering and image analysis technique we introduce here can enable rapid drug tests of metastatic phenotypes with limited cell numbers and without a requirement to expand the cells as cultures or xenografts.

The combination of tethering and image analysis allowed us, for the first time, to estimate morphological stability by measuring the time delay autocorrelation coefficients of metrics for total McTN phenotype. The larger correlations of the aggregate McTN metric in drug treatments quantify a morphological stability which is consistent with the biochemical stability known to be driven by these drugs. Previous research had shown that cells from EMT-induced cell lines have more McTNs, higher reattachment rates and embed themselves into endothelial cell layers [34]. Cell lines rich in McTNs due to tau-induced microtubule stabilization trap more efficiently in the lung capillaries of living mice [22]. While metastatic breast tumor cells are known to have higher McTN incidence, it has not been possible before now to measure McTN dynamics quantitatively. It also remains unknown whether McTNs that are increased by microtubule-stabilizing drugs, like paclitaxel, will be more efficiently trapped in lung capillaries. Our analysis here shows that paclitaxel-treated cells have longer McTNs, but less dynamic McTNs which may suggest cells would have a reduced ability to extravasate through endothelial cells and out of the bloodstream.

In this study, we developed several different tools for measuring tumor cells in a non-adherent environments including area of the cell body, variance in the cell body area, ratio of the full cell perimeter to the cell body perimeter, distance of McTN tips from cell body perimeter, number of McTN tips, cumulative tip distance, and time delay autocorrelation coefficients. These quantitative tools demonstrate an improved method for characterizing cytoskeletal phenotype in adherent cells in a non-adherent environment and have implications for understanding whether length, number, total McTN phenotype, or fluctuations in morphology are key predictors for increased reattachment of CTCs. Additionally, the tools we have developed here will enable future work exploring whether drugs that induce cells to form less dynamic McTNs are more likely to get trapped in the capillaries of distant tissues *in vivo*, as well as whether the McTNs are more or less dynamic in different breast cancer subtypes or different stages of metastatic progression.

The ability to automatically measure detailed phenotypes of the physical properties of circulating tumor cells also has potential applications in studying patient CTCs, and perhaps ultimately in supporting the selection of appropriate drug therapies for patients. Measuring McTNs is a fast assay that could give insight to cancer progression and drug response, especially since McTNs are known to reflect stem cell characteristics and epithelial-to-mesenchymal transition [35, 36]. Long-term growth of patient tumor cells in culture or as patient-derived xenografts introduces numerous potential variables and selective pressures. The significantly shorter timeframe of McTN analysis (<24h) could possibly help reduce these time-dependent pressures, since the cells were much more recently removed from the patient.

## MATERIALS AND METHODS

### Cell culture

Both human MDA-MB-436 and MDA-MB-231 cells derived from a metastatic pleural adenocarcinoma were obtained from the American Type Culture Collection and were used for all drug-treatment experiments. For experiments comparing free-floating and tethered cells, only human MDA-MB-436 cells were used. Human MDA-MB-436 and MDA-MB-231 cells were selected as a cell model for metastatic potential and presence of McTNs [29, 37]. Both cell lines were cultured in DMEM media containing 10% Fetal Bovine Serum and 1% penicillin/streptomycin. Cells were detached from cell culture plates at a confluency as close to 80% as possible using trypsin.

For drug treatments, all reagents were obtained from Sigma Aldrich and concentrations were based on previous studies. For microtubule stabilization, 1.2 μM paclitaxel was administered (10 μg/ml), and for microtubule destabilization a final concentration of 125 μM colchicine was selected. Drug concentrations were selected for non-toxicity based on previous studies [28, 35, 38, 39].

### Free-floating cells

For the experiments involved in suspended free-floating cells, ibidi microfluidics chambers were coated with 1% pluronic F-127 solution for 30 minutes. Cells were treated with a 1:10,000 dilution of CellMask-Orange (Life Technologies) membrane stain in order to visualize McTNs. Next, cells were treated with the vehicle or a drug treatment of 10 μg/mL paclitaxel or 125 μM colchicine. A 150 μL sample of treated cells was added to each ibidi channel at a concentration of 30,000 cells per channel. Cells were incubated at 37C to allow absorption of CellMask and drug treatment for 30 minutes prior to imaging.

### Tethered cells

All tethered cell experiments were conducted in 6-chamber microfluidic slides μ-Slide (Ibidi #80601) coated with five cytophobic polyelectrolyte multilayers (PEMs). Microfluidic chambers were pre-coated with.047M polyallylamine hydrochloride (Alfa Aesar #43092) for 15 minutes in order for the PEM layers to stick. In each PEM, 5 minutes of anionic polymer.01M polymethacrylic acid (Polysciences #00578) followed by cationic polymer polyacrylamide (Polysciences #02806) was applied for 5 minutes. Finally, the addition of lipid moiety N-[1-(2,3-Dioleoyloxy)propyl]-N,N,N-trimethylammonium methyl-sulfate (DOTAP, Avanti #890890) was administered for 5 minutes. All polymer and lipid solutions had pH 3.0. In order to cross-link the DOTAP to the substrate, an additional 5-minute step of 3.7% formaldehyde was applied.

After each polymer, lipid, or formaldehyde treatment, microfluidic chambers were washed with 2 one-minute washes of deionized water at pH 3.0. Cells received the same treatment of CellMask-Orange and drugs as free-floating cells.

### Confocal microscopy

All imaging was conducted on an Olympus FV-1000 confocal at a 60x magnification.

For videos of suspended cells, a set of five 0.5 μm/slice z-stacks were imaged every 6.5 seconds for a total time series of 20 z-stacks. For tethered cells, z-stack slices were 1μm thick and stacks were imaged every 10 seconds. In all cases, the middle z-slice was calibrated along the z-axis to where the cell appeared largest.

### Image analysis

For each time point, a max intensity image of each z-stack was computed, all further processing was derived from max intensity images. Outlines for the cell body and full-cell were computed separately. Cell body outlines were identified by using image analysis methods published previously [30]. For drug treatment experiments, cells with cell body centroids migrating more than 5 μm were excluded from further analysis.

In order to get clear outlines of the McTN features, we modified and combined previously published image analysis techniques optimized for cell shape along with techniques optimized for stress fibers by using a rotating anisotropic filter [30] [31]. Consequently, analysis for full cell outlines processed and optimized parameters for 3 distinct cellular regions separately: McTNs, bright cell body border and globular base of protrusion region, and the cell center. The full cell outline was derived from a binary image comprised of the 3 distinct analyses (Supplementary Figure 1).

#### First analysis

Due to the fact that McTN features were significantly dimmer than the cell body and filamentous rather than globular, the first analysis was optimized specifically for the McTNs. First, a 2 x 2 median filter was applied to the maximum z-projection per time-point in order to give a very fine-featured, localized smoothing optimized for approximately half the width of McTNs (Supplementary Figure 2A). Following, the output underwent an initial rough convolution with a rotating anisotropic filter that will be described in more detail below (Supplementary Figure 2B). After the initial iteration of rotating anisotropic filtering was applied, contrast adjustment algorithms in matlab were optimized to make the protrusions rather than cytoplasm uniformly white (Supplementary Figure 2C). Additionally, to specifically extract the McTN features, the output underwent another iteration of rotating anisotropic filtering; multiple iterations of the rotating anisotropic filtering were repeated using the combined output of the previous series of filtering (Supplementary Figure 2D) [31]. The 60 anisotropic filters were computed by convolving a Laplacian kernel with 60 different Gaussian kernels aligned at different angles. The contrast adjusted image (Supplementary Figure 2C) were convolved with the 60 anisotropic filters optimized for 60 different angles. Each individual anisotropic filter emphasizes alignment along a different angle. Next, the max projection of all 60 anisotropic filter results was computed and underwent a pass of contrast adjustments using built-in MATLAB function imadjust weighted for optimizing McTN brightness. The contrast adjusted image next underwent several iterations of rotating anisotropic filtering followed by a max intensity projection across all angles. Finally, the resulting anisotropic results were linearly multiplied with the initial rotating anisotropic results before the contrast was adjusted again and then thresholded (Supplementary Figure 2E and 2F) Because the rotating anisotropic filter selects preferentially for line-like features occasionally truncating, rather than intersecting with the cell body or potentially incorrectly biasing cell body curvature near the base of the protrusion, a second analysis optimized for features including base of the McTNs and near the cell body boundary was conducted independently.

#### Second analysis

For this second region, the initial image underwent a matched filtering technique originally designed for retinal segmentation followed by Otsu thresholding (Supplementary Figure 1B) [40, 41]; this technique showed preference for the base of the protrusions. The retinal segmentation technique was image multiplied with the initial anisotropic filtering results prior to being an input into the previously establish local, global curvature technique [30].

#### Third analysis

Lastly, to prevent the analysis optimized for tentacles from creating an annular outline, a rough estimate of the cell center was computed by using the matlab built in function of imfilter to blur the image, thresholding, and then using matlab’s bmorph to remove any spurs and erode shape to prevent it to contributing to cell outline information (Supplementary Figure 1C). Cell center analysis did not require any contrast optimization.

#### Composite analysis

Once all 3 analyses were complete, results were added together for a composite binary image (Supplementary Figure 1D) and built-in morphological operations in MATLAB were used to remove the image from any small noise generated objects (Supplementary Figure 1E). Finally, the boundary derived from the composite binary image was inputted into an active contour algorithm as an initial estimate (Supplementary Figure 1F) [42, 43].

Once the outline of the full cell perimeter was computed, the tips were computed by first finding continuous positive regions from the binary McTN image. From the images’ continuous positive segments, tips were selected by locating the coordinates outside the cell body boundary of maximum local curvature.

Attributes derived from image analysis consisted of McTN inclusive outline of the full cell, outlines of the cell body exclusively, centroid of cell body, and the tips of the McTNs. From these attributes, measurements of McTN behavior were derived including, area of the cell body, variance in the cell body area, total distance traveled by the centroid of the cell body boundary, ratio of the full cell perimeter to the cell body perimeter, distance of McTN tips from cell body perimeter, number of McTN tips. Additionally, cumulative tip distance was measured by multiplying the number of McTNs by the average distance of McTN tips from the cell body boundary per frame per cell. All attributes and metrics were computed in matlab. An executable file that does not require matlab is freely-available for download at the following address: http://innovatetech.com/cellthsystems-software.

### Statistics

All statistics results were measured in matlab. Normalness of data distribution was tested for a skewness of ±2 or a kurtosis between 0-6. For normally distributed data, a standard t-test was conducted for 2-sample comparison (tethered verses suspended) and ANOVA analysis for multi-sample comparisons (drug studies). For non-normally distributed data, a Kolmogorov-Smirnov test was conducted as an additional check.

## SUPPLEMENTARY MATERIALS FIGURES



## Abbreviations

CTC: circulating tumor cells
EMT: epithelial-to-mesenchymal transition
McTN: microtentacle
DMEM: dulbecco’s modified eagle medium
CSC: cancer stem cell
