# Supplementary material for: Extracting microtentacle dynamics of tumor cells in a non-adherent environment
Source: Oncotarget. 2017 Dec 4;8(67):111567–80. doi: 10.18632/oncotarget.22874 (PMC5762343; doi:10.18632/oncotarget.22874)
Supplement: Supplementary file 1 [file oncotarget-08-111567-s001.pdf]

Extracting microtentacle dynamics of tumor cells in a non-adherent environment

SUPPLEMENTARY MATERIALS

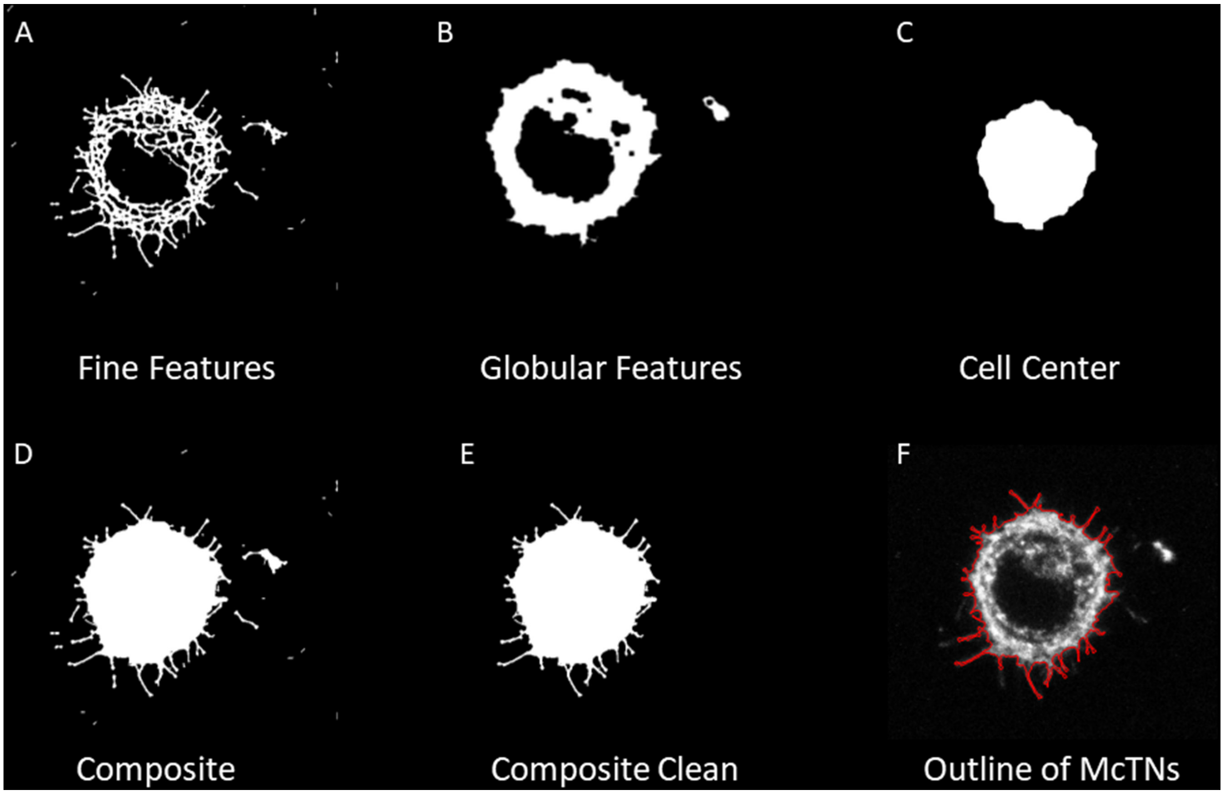

Supplementary Figure 1: Full cell outline is the composite of analyses optimized for 3 distinct cellular regions. (A) Binary image results for algorithms optimizing for fine featured McTNs. **(B)** Binary image results for algorithms optimizing for globular features. **(C)** Binary image results for computing a rough cell center. **(D)** Binary image composite of 3 binary results from different cell regions. **(E)** Binary image composite cleaned of debris. **(F)** Outline overlay of final results on initial image includes McTNs.

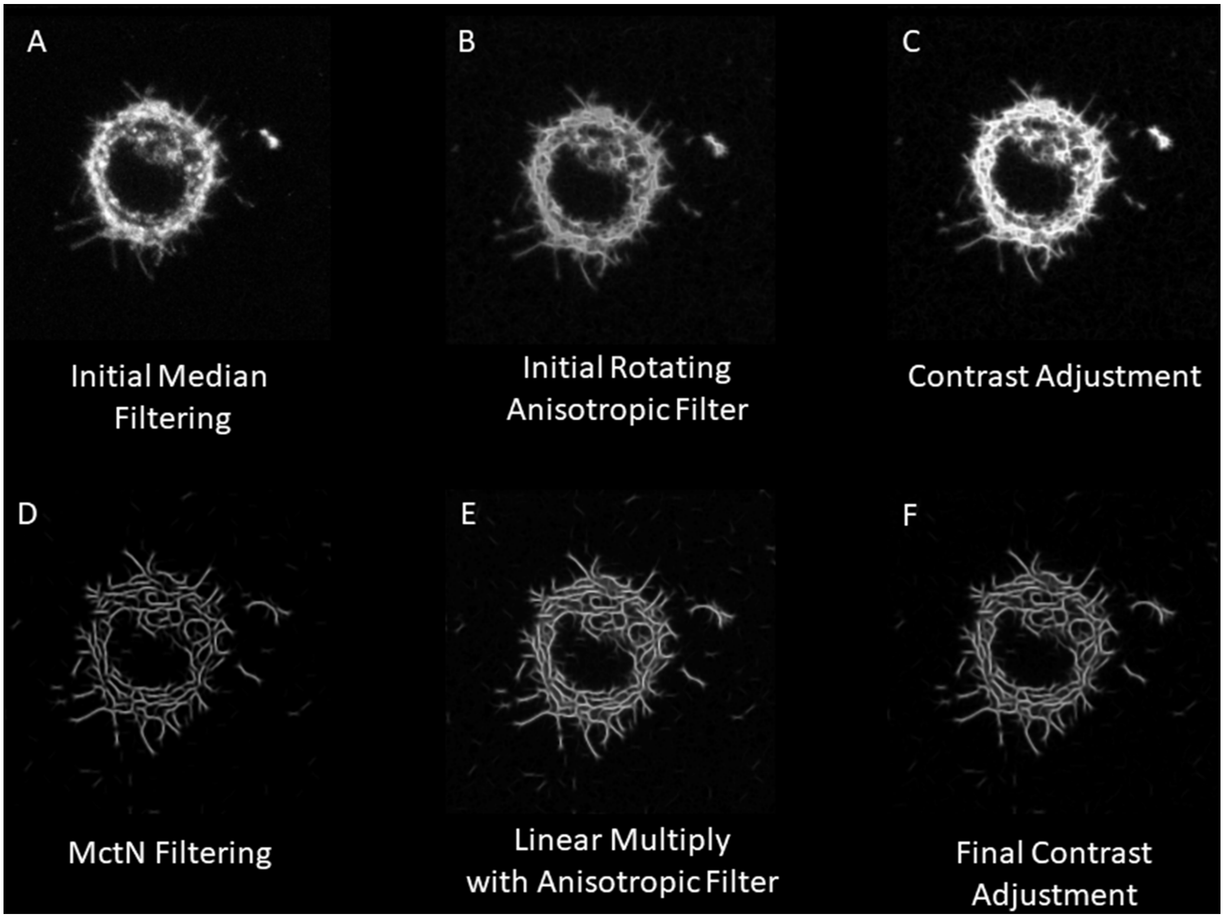

Supplementary Figure 2: Results of processing steps optimizing for fine featured microtentacles. **(A)** Median filter was applied to the maximum z-projection results. **(B)** Results from first convolution with a rotating anisotropic filter routine. **(C)** Contrast adjustment results optimizing contrast for fine-featured tentacles. **(D)** Results from multiple iterations of a rotating anisotropic filter. **(E)** Results from linear multiplication between intial and final rotating anisotropic filter results. **(F)** Final contrast adjustment results prior to thresholding.

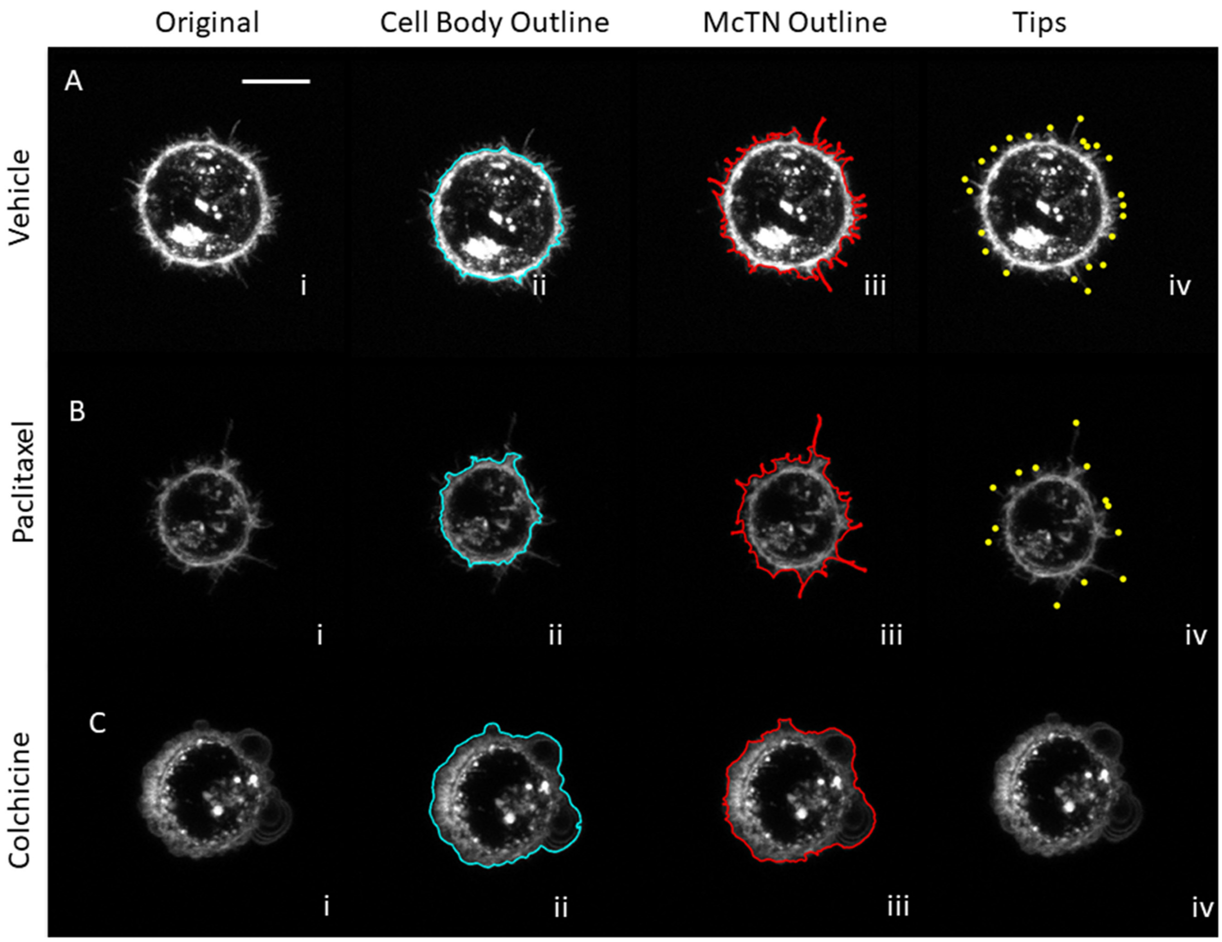

Supplementary Figure 3: Image analysis attributes for microtubule-targeting drug treatments for MDA-MB-231 cells. **(A)** Max projection of z-stack for MDA-231 cells treated with vehicle (i) is analyzed to find cell body boundary (ii), outline of full cell, (iii) and tips of McTNs (iv). **(B)** Max projection of z-stack for MDA-231 cells treated with 10 µg/mL paclitaxel (i) analyzed for cell body boundary (ii), outline of full cell, (iii) and tips of McTNs (iv) shows increase in McTNs. **(C)** Max projection of z-stack for MDA-231 cells treated with 125 µM colchicine (i) analyzed for cell body boundary (ii), outline of full cell, (iii) and tips of McTNs (iv) shows a decrease in McTNs (scalebar = 10µm).

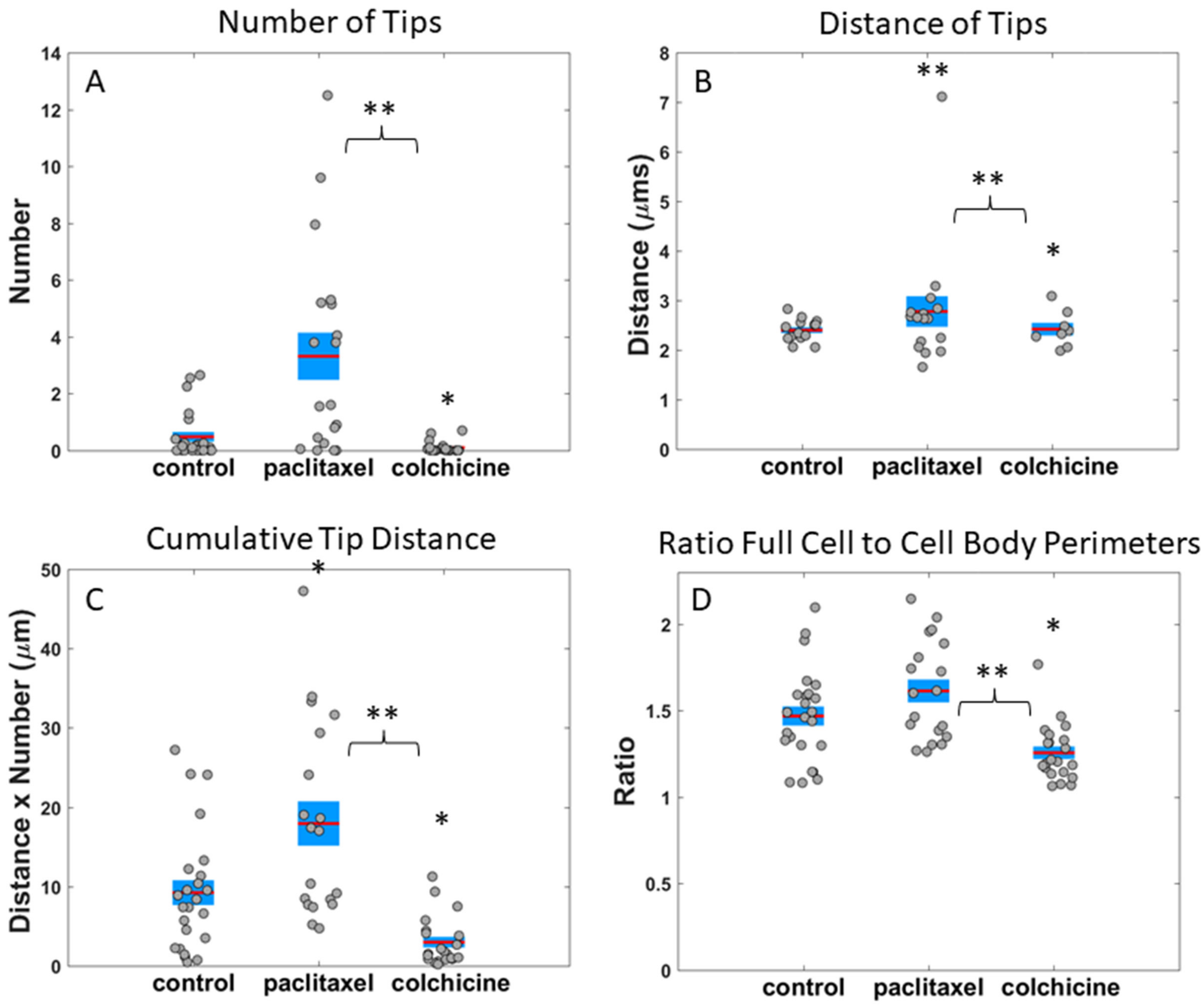

Supplementary Figure 4: Measurements of microtentacle attributes in MDA-MB-231 cells for different drug treatments. **(A)** Average number of McTN tips for cells treated with vehicle, 10 µg/mL paclitaxel, and 125 µM colchicine. **(B)** Average distance of McTN tips from cell body boundary for cells treated with vehicle, 10 µg/mL paclitaxel, and 125 µM colchicine. **(C)** Average cumulative tip distance, calculated by multiplying total number of McTN tips by the average distance of McTN tip from cell body per frame, is shown for cells treated with vehicle, 10 µg/mL paclitaxel, and 125 µM colchicine. **(D)** Ratio of perimeters for full cell outline to cell body boundary is shown for cells treated with vehicle, 10 µg/mL paclitaxel, and 125 µM colchicine. Horizontal bar represents average across cells; shaded area, SEM; and individual dots, mean value per cell across time series. *P<0.05; **P<0.001 ANOVA test.

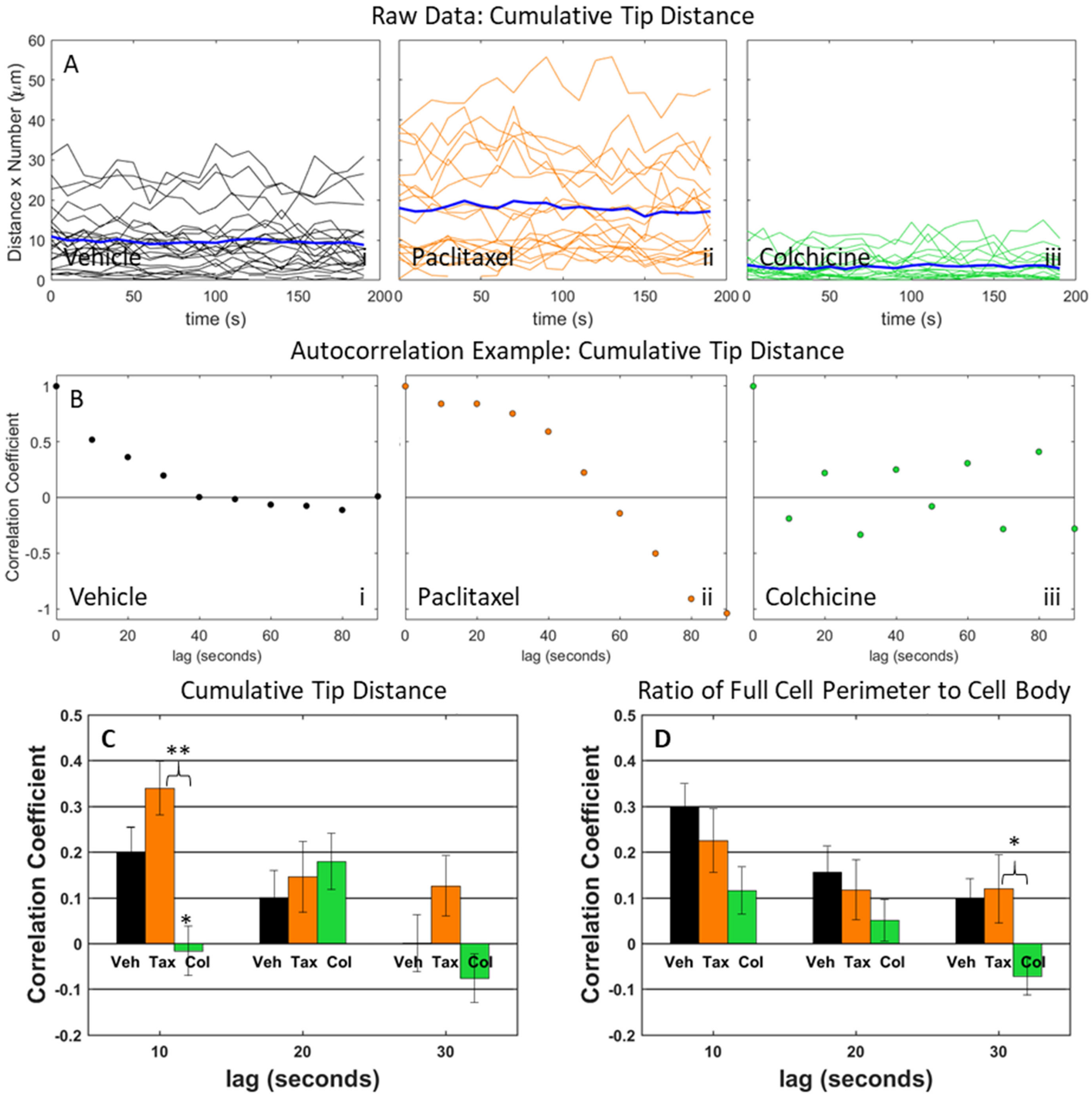

Supplementary Figure 5: Dynamic behavior in MDA-MB-231 cells is assessed by analyzing cumulative tip distance and the ratio of full cell perimeter to cell body perimeter. **(A)** Time traces or cumulative tip distance for individual cells treated with vehicle (i), 10 μg/mL paclitaxel (ii), and 125 μM colchicine (iii). Bold blue time trace is average cumulative tip distance over all individual cells. **(B)** Example autocorrelation traces of cumulative tip distance for individual cells treated with vehicle (i), 10 μg/mL paclitaxel (ii), and 125 μM colchicine (iii). **(C)** Fluctuations of cumulative distance is shown by computing the autocorrelation coefficient at time lags 0 to 30 seconds for cells treated with vehicle, 10 μg/mL paclitaxel, and 125 μM colchicine. **(D)** Fluctuations of ratio between full cell outline and cell body boundary is shown by computing the autocorrelation coefficient at time lags 0 to 30 seconds for cells treated with vehicle, 10 μg/mL paclitaxel, and 125 μM colchicine.
